# Evaluation of the Effects of Electrical Stimulation: A Pilot Experiment on the Marine Benthic Foraminiferal Species *Amphistegina lessonii*

**DOI:** 10.3390/life13040862

**Published:** 2023-03-23

**Authors:** Federica Rebecchi, Davide Lattanzi, Sigal Abramovich, Patrizia Ambrogini, Caterina Ciacci, Michele Betti, Fabrizio Frontalini

**Affiliations:** 1 Department of Pure and Applied Sciences, Urbino University, 61029 Urbino, Italy; fabrizio.frontalini@uniurb.it; 2 Department of Biomolecular Science, Urbino University, 61029 Urbino, Italy; davide.lattanzi@uniurb.it (D.L.); patrizia.ambrogini@uniurb.it (P.A.); caterina.ciacci@uniurb.it (C.C.); michele.betti@uniurb.it (M.B.); 3 Department of Earth and Environmental Sciences, Ben Gurion University of the Negev, Beer Sheva 84105, Israel; sigalabr@bgu.ac.il

**Keywords:** pollution, pseudopodial activity, impact, bioindicator

## Abstract

Environmental disturbances resulting from anthropogenic energy pollution are intensely growing and represent a concern for the marine environment. Benthic organisms are the significant fauna exposed to this kind of pollution; among them, foraminifera are largely used as pollution bioindicators in marine environments, but studies on the effects induced by electrical stimulation are not documented. In the present research, we evaluated the effects of short-term different electric current densities on the viability of benthic foraminiferal species *Amphistegina lessonii* by checking the pseudopodial activity and defined the threshold electrical density range. After 3 days of treatment, *A. lessonii* stimulated with a constant current showed pseudopodial activity at a lower electric current density (0.29, 0.86 μA/cm^2^) up to 24 h. With increasing stimulation time, the percentages of pseudopodial activity decreased. The pseudopodial activity was absent at high current densities (5.71, 8.57 μA/cm^2^). The viability of *A*. *lessonii* exposed to a pulsed current was higher at a low and middle electric current density (from 0.29 to 5.71 μA/cm^2^) than at a high electric current density (from 11.43 to 20 μA/cm^2^). Based on these preliminary results, the selected benthic foraminiferal species seems to better stand pulsed currents than constant ones. These first experiments might provide useful information for the definition of the appropriate electrical density threshold to avoid side effects on a part of the benthic community.

## 1. Introduction

The impacts of human activities on natural resources, diversity and ecosystem functioning have reached a critical level in recent decades and are even expected to worsen due to the current rate of climate change [1]. Marine pollution does not only involve chemical substances, both organic and inorganic, released into the marine environment, but it also embraces different forms, such as energy input. This form of pollution can directly or indirectly affect the integrity of the marine ecosystem, its functioning and the biota living therein. Environmental disturbances that result from anthropogenic energy pollution are intensely growing and represent a great concern for the marine environment [2]. Indeed, the Marine Strategy Framework Directive (MSFD 2008/56/EC) sets out 11 qualitative descriptors for characterizing the marine environment in terms of its “Good Environmental Status”. Among them, the aim of descriptor 11 is that the “*Introduction of energy, including underwater noise, is at levels that do not adversely affect the marine environment*” [3].

Anthropogenic sources of energy in the marine environment can be commonly ascribed to: (a) offshore operations, wind turbines, as well as submarine power cables that generate both electric and electromagnetic fields and (b) the construction of infrastructure, the use of sonars, shipping for trade or tourism, dredging and military activities that altogether are responsible for generating underwater noise [3]. About 70% of submarine cables are in European sea waters and supply electricity to islands and oil platforms, interconnect countries or transfer electricity from marine renewable energy devices [4]. The installation, maintenance and discharging phases of submarine cables may cause environmental effects on marine life and the surrounding habitats [5]. The equipment used for cable preparation may induce physical disturbances, such as the alteration of the substratum, leading to the direct destruction of benthic habitats, flora and fauna. Consequently, substratum alteration may cause displacement and damage to organisms, depending on the composition of the benthic community and the sensitivity and resilience capability of the affected species or communities [5]. Sediment resuspension is a further consequence of sediment reworking, which leads to suspended particulate matter concentrations. Different studies have highlighted how resuspended material may decrease the water transparency, with effects on primary producers; the feeding ability of fish, which visually detect their prey [6]; the efficiency of invertebrate filter feeding [7,8] and gill damage in young fish larvae [9]. In addition, sediment resuspension may release pollutants buried in the sediment, particularly those located in coastal areas that are affected by human activities [5]. Furthermore, the electrodes can release toxic electrolysis products (chlorine and bromine), which can be harmful to marine organisms and affect the overall quality of marine waters [10], or heavy metals (e.g., Cu, Al, Pb, Zn) can dissolve into the sediment from damaged and abandoned cables [5]. Heat emission may occur when electric energy is transported, leading to an increase in the temperature at the cable surface and in the surrounding environment [11]. Temperature radiation can also cause physiological changes in benthic organisms [11].

Recently, environmental concerns related to submarine cables have been raised owing to the production of electromagnetic fields in marine environments [5]. The submarine cable systems facilitate the transfer of electrical currents, which may be either alternating current (AC) or direct current (DC) [4]. The current flow passing through power cables generates electromagnetic fields that can be divided into electric and magnetic fields [4]. The strengths of both the magnetic and electric fields rise with the current flow and decrease with the distance from the cable [5]. The electric field can be induced by water movement [12] or by any movement of organisms through the geomagnetic field [13]. Marine organisms are also surrounded by alternating currents and direct electric fields called bioelectric fields [14]. An artificial electric field can be generated during the transfer of electric currents between two electrodes placed on the seabed, which may affect the natural fields near the cables or create an electromagnetic field that may be harmful to marine organisms living close to it [15].

All benthic and demersal species are exposed to such artificial sources, and their sensitivity to electric fields is specific to the considered species [16]. Marine species such as elasmobranchs, fishes, mammals, turtles, molluscs and crustaceans have highly electrosensory organs devoted to predation, mate recognition, migration and orientation in the Earth’s magnetic field [4,12]. This natural sense can be altered by the electric fields emitted by a submarine transmission system [15]. Elasmobranchs, the group of marine animals most sensitive to electric fields, may detect very low electric fields (from 0.005 to 0.02 μV cm^−1^) [17,18], and may be repelled by high electric fields of 400 and 1000 μV m^−1^ [19,20]. The strong electric field produced by a cable acts as a barrier, preventing movement between important areas (such as feeding, mating and nursery areas) [12]. The response of elasmobranchs to submarine cables is species-specific, and may even be individual-specific, as demonstrated in mesocosm studies [21]. Similarly, teleost fishes are sensitive to electricity, in fact they may be repulsed by strong electric fields of 6–15 V m^−1^. On the other hand, the anguillidae are sensitive to weak electric fields ([12] and references there in). Furthermore, no changes in physiology or survival have been observed in the teleost fish [22].

Surprisingly, the impact of artificial electric currents on invertebrates has been poorly studied [5]. The effect of an electric field has been reported to induce freshwater crayfish behavioral modifications [23,24] or a reduction in body motion [25]. Moreover, American lobsters showed a slight change in behavioral activity [26]. A complete and exhaustive list of studies that examined the effect of electromagnetic fields on marine invertebrate species can be found in Albert et al. [4]. However, these studies evaluated only the effect of the anthropogenic magnetic field, while a summary of studies that evaluated the electric field is provided in Table S1 [17,18,19,20,22,23,24,25,26,27,28,29,30,31,32,33,34,35,36,37,38,39,40,41]. The development of biological indicators as a tool to evaluate the health of ecosystems has been devoted to assessing the ecological quality status of aquatic environments [42]. Benthic invertebrates represent a reliable biological indicator for the definition of the ecological quality status of marine ecosystems [43]. Among benthic fauna, foraminifera have been widely applied in biological monitoring [44]. Benthic foraminifera are mainly marine single-celled organisms, with their cell body commonly enclosed in a shell (also known as test) [45]. They are the most varied and abundant protozoa in the marine realm compared to other shelled microorganisms [46]. They inhabit all kind of environments, from marine to transitional marine ecosystems, and play a significant role in the biogeochemical cycle of organic and inorganic compounds [45]. They are also abundant and easy to collect and analyze, making them suitable for statistical analysis, even in the case of reduced sediment availability [47]. Due to their mostly short life and reproductive cycles, they are highly sensitive to any environmental change and respond rapidly to natural and anthropogenic alterations, making them an early-warning tool for environmental monitoring assessment [48].

The genus *Amphistegina*, selected for this study, is a symbiont-bearing benthic foraminifera largely used as a bioindicator of water quality because of its high sensitivity to the physical characteristics of seawater [49]. Turbidity of water and photo-inhibitory stress related to high water temperatures may lead to the mortality of *Amphistegina*’s symbionts and consequently bleaching in *Amphistegina* [49]. Additionally, *Amphistegina* is considered as an important element for the calculation of the biotic index (i.e., FoRAM Index [50,51]) that is used to evaluate environmental conditions in a coral reef environment. This genus hosts diatom symbionts [52] and is common and abundant in tropical and subtropical reefs, where it contributes to carbonate production [53] and to the stability of global reef habitats [54]. Strongly dependent on water temperatures, they have been reported in the Pacific and the Atlantic Ocean and in the Red and the Mediterranean Sea [55]. This genus has rapidly expanded its biogeographic range towards higher latitudes [53,56]. Currently, five species of *Amphistegina* (*Amphistegina bicirculata, A. lessonii, A. lobifera, A. papillosa* and *A. radiata*) are present in the Red Sea [57]. The massive migration event of *Amphistegina*, termed Lessepsian migration, has allowed the rapid colonization of this genus in the Mediterranean Sea. At the present, only two species (*A. lessonii* and *A. lobifera*) have been recorded in the Mediterranean Sea [53]. The biomarkers of *A. lessonii* have also been used to evaluate the water quality in the Fernando de Noronha Archipelago in Brazil [58]. More recently, significant changes in the cell biochemistry (e.g., increases in lipid peroxidation, metallothionein-like protein and total SOD activity) of *A. lessonii* were documented in response to Zn exposure [59]. Similarly, it was revealed that exposure to Hg leads to marked variations in the biochemistry of *A. lessonii* which are mainly associated with oxidative stress (i.e., the production of reactive oxygen species), including the depletion of glutathione and changes in the synthesis of protein [60]. The observed biochemical changes in this species in response to pollutants (i.e., Hg and Zn) have therefore been suggested as a potential way to detect early evidence of environmental stress in biomonitoring.

To date, no study has documented the effects induced by electrical currents on benthic foraminifera. Therefore, this in vivo experiment aims to evaluate the effects of short-term different electric current densities on the viability of benthic foraminiferal species *Amphistegina lessonii*, and to define the threshold electrical density range.

## 2. Materials and Methods

### 2.1. Collection of Individual Specimens

Living specimens of A. lessonii were collected from rock pebbles at Eilat in the Gulf of Aqaba (Red Sea, Israel) from June to September 2022. The adult living individuals (ca. 300–600 μm) were placed in 50 mL Falcon tubes and then transferred to the Micropaleontological Laboratory at the University of Urbino (Italy). Once in the laboratory, the individuals were placed in 100 mm glass Petri dishes with natural seawater with a salinity of 40 for acclimatization at 25 °C with 12:12 h light and dark cycles for several days. Only the living specimens of A. lessonii with a clear golden-brown color and exhibiting evident pseudopodial activity were selected for the present experiment.

### 2.2. Development of the Electric Generator Prototype

**Figure 1:**
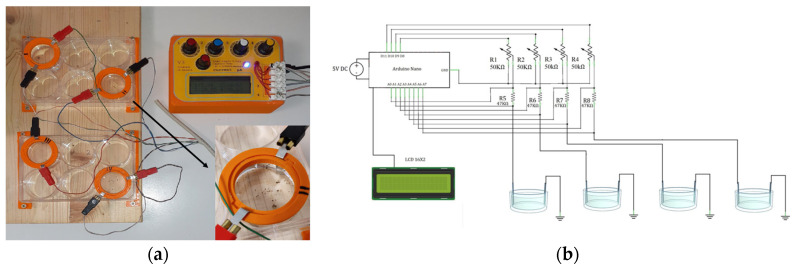
Electrical stimulator specifically designed for the foraminiferal (i.e., *Amphistegina lessonii*) experiment. (**a**) Picture of stimulus device that is connected to four pairs of electrodes and a section of the well. (**b**) Wiring diagram of the stimulator.

Living foraminiferal specimens were electrically stimulated using a stimulus generator made up of one Arduino Nano open-source electronic prototyping board (Arduino, Italy) based on the ATmega328 microcontroller. The Arduino board has been programmed using the Arduino open-source integrated development environment (IDE) and was used to generate constant or pulsed low-intensity current stimulation directly from digital channels. The intensity of current stimulation was measured using analog input channels. The board was connected to LCD 16 × 2 to visualize the current stimulus intensity. The rectangular flat electrodes were made up of platinum (dimensions: 4 mm in width, 0.2 mm in thickness) and were placed in a multiwell plate (i.e., UltraCruz^®^ Tissue Culture six wells sterile plate), each filled with 9.6 mL of artificial seawater (prepared in accordance with the composition indicated in ASTM D1141-98 [61]), and immersed to a depth of about 1 cm (Figure 1a). Each Arduino board was able to control four pairs of electrodes. The chip pins D8, D9, D10 and D11 were connected to positive platinum electrodes (anodes), each through 50K potentiometers in a series with 47K resistors (Figure 1b). The value of the potentiometer and the resistance have been chosen to obtain a current range from 0 to 100 μA. The chip pins A1–A7 were connected two by two across the 47K resistors to calculate the current flow (Figure 1b). The 50K potentiometers were used to fine-tune the current intensity. Finally, the negative electrodes (cathodes) were connected to the Arduino ground. Before each experiment, the stimulation current was measured with a commercial multimeter to verify that it was equal to that calculated by the Arduino board. A comprehensive part list and the wiring diagram are shown in Figure 1b and the Arduino code for the constant and pulsed stimulation is provided in the Supplementary Materials S1 and S2.

### 2.3. Experimental Setup

The experimental procedure consisted of a measurement of the viability of *A. lessonii* specimens after exposure to both constant and pulsed direct current stimulation. The foraminiferal specimens were stimulated with the following constant current values 0 (control), 1, 3, 5, 10, 20 and 30 μA, that are equivalent to 0, 0.29, 0.86, 1.43, 2.86, 5.71 and 8.57 μA/cm^2^, respectively, (0.7–1.12 V); and the following pulsed current values 0 (control), 1, 3, 5, 10, 20, 30, 40, 50, 60 and 70 μA, that are equivalent to 0, 0.29, 0.86, 1.43, 2.86, 5.71, 8.57, 11.43, 14.29, 17.14 and 20 μA/cm^2^, respectively, (0.08–1.32 V). The electrical current density (μA/cm^2^) was used to quantify the effects of the electric current, as it describes the amount of electric current flowing per unit of the cross-section area of the plate. A total of 850 living individuals of *A. lessonii* were selected under the optical microscope and 10 of them were randomly placed in a six-well plate and exposed to each value of the electric current intensity. The experiments were carried out over a period of 3 days and each treatment consisted of five replicates. After the exposure, the viability was evaluated after 24, 48 and 72 h by checking the pseudopodial activity. The individuals that clearly exhibited a stream of pseudopodia were counted as living, all the others were counted as non-living individuals. The absence of evidence of pseudopodial activity cannot, however, be directly related to the mortality of the foraminiferal specimens; in fact, the cytoplasm can be retracted within the test, even under adverse conditions (i.e., stress) [62,63].

### 2.4. Statistical Analysis

The Kruskal–Wallis H’ test is a non-parametric test and was used to check if there were significant differences among the samples (i.e., control and treatment samples). This test was then followed by a post-hoc Dunn’s test for the specific sample pair comparison. The confidence levels were reported at 99.9%, 99% and 95% (that is α = 0.001, 0.01, 0.05). The half maximal effective concentration (i.e., EC50) parameter was then calculated for both the constant and pulsed current and at time exposures, namely 24, 48 and 72 h. The EC50 is here used to define the maximum electrical current density (μA/cm^2^) to exert half of its maximal response.

## 3. Results

**Figure 2:**
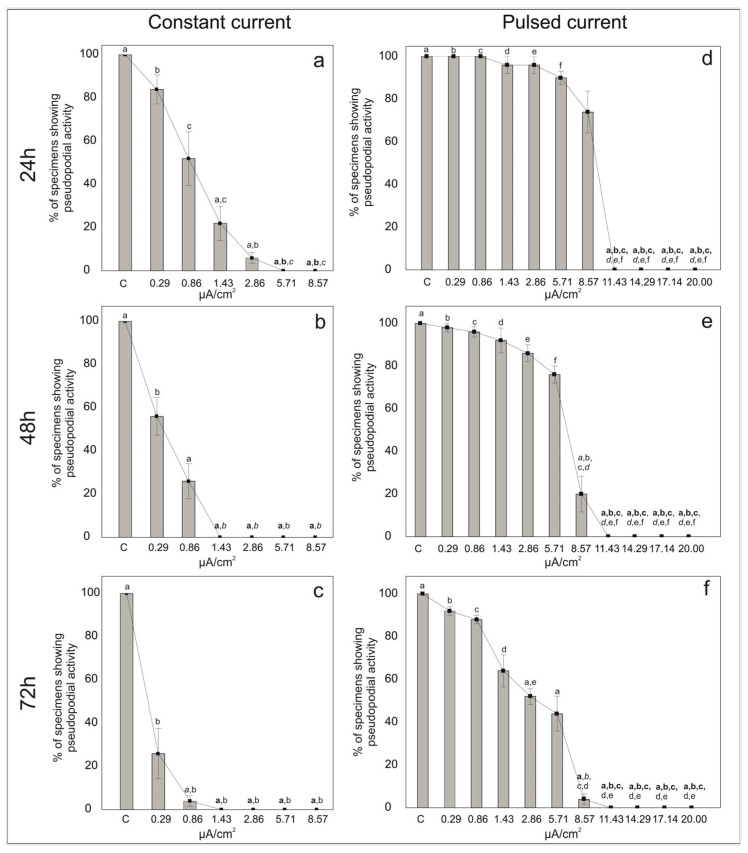
Percentages of living individuals showing pseudopodial activity in the symbiont-bearing foraminiferal species *Amphistegina lessonii* in samples treated with constant (**a**–**c**) and pulsed (**d**–**f**) current for 24 h (**a**,**d**), 48 h (**b**,**e**) and 72 h (**c**,**f**) and in control samples (no current). Data are reported as mean ± standard deviation (*n* = 5). Letters denote significant differences (bold *p* < 0.01, italic *p* < 0.01 and *p* < 0.05) between the different conditions as revealed by the post-hoc Dunn’s test for the specific sample pair comparison.

After 3 days of stimulation, the percentage of individuals that clearly exhibited a stream of pseudopodia were counted as living, all the others were counted as non-living individuals. With the increasing electric current density of both the constant and pulsed current, the percentage of individuals showing pseudopodial activity decreased (Figure 2 and Table S2).

### 3.1. Constant Current

In the control treatments of the constant current, all the individuals of *A. lessonii* showed pseudopodial activity. At low electric current densities, 84% of foraminifera exhibited pseudopodial activity at 0.29 μA/cm^2^ and 52% of specimens were still active at 0.86 μA/cm^2^ after 24 h of exposure (Figure 2a). However, with increasing stimulation time (i.e., 48 h and 72 h), the percentages of pseudopodial activity decreased (Figure 2b,c). For electric current densities of 1.43 μA/cm^2^ and 2.86 μA/cm^2^ the percentage of individuals with pseudopods decreased to ca. 22% and 6% after 24 h, respectively, (Figure 2a) and no specimens showed pseudopodial activity after 72 h. The pseudopodial activity was even absent at quite high currents densities, namely 5.71 and 8.57 μA/cm^2^ just after 24 h.

The EC50 at 24, 48 and 72 h were calculated and corresponded to 0.87, 0.37 and 0.18 μA/cm^2^, respectively. On the basis of these data, the Kruskal–Wallis H’ test indicated that there were significant differences among the groups χ^2^(6) = 30.33, *p* < 0.001 at 24 h; χ^2^(6) = 31.96, *p* < 0.001 at 48 h and χ^2^(6) = 28.2, *p* < 0.001 at 72 h. The post-hoc Dunn’s test indicated that the mean ranks of several pairs were significantly different (Table S3). Specifically, the control, 0.29 and 0.86 μA/cm^2^ groups showed significantly different results to the 1.43, 2.86, 5.71 and 8.57 μA/cm^2^ groups at 24 and 48 h (Table S3). No significant differences were found between the control, 0.29 and 0.86 μA/cm^2^ groups at 24 and 48 h, but the 0.86 μA/cm^2^ condition was different from the control and 0.29 μA/cm^2^ groups after 72 h of exposure (Table S3).

### 3.2. Pulsed Current

In the control treatments of pulsed current, all the individuals showed pseudopodial activity throughout the experiment (i.e., up to 72 h). The percentages of specimens with pseudopodial activity were high at low and middle (i.e., up to 5.71 μA/cm^2^) current densities at least up to 24 h of exposure (Figure 2d). After 72 h at 0.29 μA/cm^2^, about 95% of the foraminifera still exhibited pseudopodial activity. With increasing current densities at 72 h, 85% of the individuals were active at 0.86 μA/cm^2^, 60% at 1.43 μA/cm^2^, 53% at 2.86 μA/cm^2^ and 37% at 5.71 μA/cm^2^. For the electric current density of 8.57 μA/cm^2^, the percentage of individuals emitting pseudopods decreased from 74% at 24 h to 4% after 72 h of exposure (Figure 2f). The pseudopodial activity was absent at very high currents densities (11.43, 14.29, 17.14 and 20 μA/cm^2^), even after 24 h of exposure.

On the basis of these endpoints, the EC50 was set at 8.96, 7.06 and 0.42 μA/cm^2^ at 24, 48 and 72 h, respectively. The Kruskal–Wallis H’ test suggested significant differences among the groups χ^2^(10) = 49.3, *p* < 0.001 at 24 h; χ^2^(10) = 49.94, *p* < 0.001 at 48 h and χ^2^(10) = 51.83, *p* < 0.001 at 72 h. Indeed, significant differences were recognized among the pair groups (Table S3). On the basis of the post-hoc Dunn’s test for the specific sample pair comparison, significant differences were observed for the control, 0.29, 0.86, 1.43, 2.86 and 5.71 μA/cm^2^ conditions and for current densities higher than 8.57 μA/cm^2^ after 24 h of exposure (Table S3). After 72 h of exposure, no significant differences were found between the control, 0.29, 0.86 and 1.43 μA/cm^2^ conditions (Table S3)

## 4. Discussion

In the present research, the short-term (up to 72 h of exposure) effects of different direct electric current densities on the viability of *A. lessonii* were evaluated by observing the pseudopodial activity. Currently, the absence of an accurate method to assess the vitality of foraminifera makes it difficult to distinguish between living and dead specimens. Different methods to check the viability of foraminifera have been proposed and applied, such as the use of terminal dyes (e.g., rose Bengal or Sudan Black B) that are, however, unsuitable for a reliable evaluation of the short-term effect [64,65]. Rose Bengal, for example, has been widely applied as a stain to distinguish living from dead benthic foraminiferal specimens and has been extensively used in field studies [64,65]. According to Bernhard et al. (2006) and Frontalini et al. (2018) [64,65], this non-vital stain might result in a marked overestimation of the abundance of living specimens by including false positive results (i.e., stained remaining proteins but not living specimens). To overcome this problem, several fluorescent probes (e.g., CellTrackerTM Green CMFDA, CellHunt Blue CMHC) have been proposed to check the viability of foraminiferal cells [66,67] and are considered a more accurate viability method [63]. Despite the recent development of these fluorescent probe-based methods, which have been shown to be suitable for determining responses to short-term disturbances (e.g., [63,65,68]), pseudopodial activity has been suggested as the most practical and the more reliable method for assessing the viability of foraminifera [62].

This study has been undertaken to test the effect of both constant and pulsed direct currents on the viability of foraminifera after 24 h, 48 h and 72 h of exposure. After 3 days of stimulation, the pseudopodial activity in *A. lessonii* seems to be negatively affected by both constant and pulsed direct currents at different electrical current densities. Although *A. lessonii* specimens seem to stand only the lowest constant current densities (i.e., 0.29 and 0.86 μA/cm^2^), the specimens with pseudopodial streaming are rather low at 24 h. Increasing the constant current densities further negatively affects the specimens of *A. lessonii* by altering their pseudopodial activity over time (i.e., 48 and 72 h). The test for foraminiferal specimens appears somewhat empty (i.e., devoid of cytoplasm) and with a whitish coloration. On the other hand, at all low and medium current densities (i.e., 0.29, 0.86, 1.43, 2.86, 5.71 and 8.57 μA/cm^2^) of pulsed current, foraminiferal specimens show pseudopodial activity. This suggests that pulsed current had a less negative impact on *A. lessonii*. No evidence of pseudopodial activity was, however, found at the highest current densities (i.e., 11.43, 14.29, 17.14 and 20 μA/cm^2^).

These different trends for constant and pulsed currents are well supported by the half maximal effective concentration (EC50) that defines higher density values for pulsed (i.e., 8.96, 7.06 and 0.42 μA/cm^2^ at 24, 48 and 72 h, respectively,) than constant current (i.e., 0.87, 0.37 and 0.18 μA/cm^2^, at 24, 48 and 72 h, respectively). This research presents the first direct evaluation of the effects of electric current densities on a foraminiferal species; therefore, there are no available data for a direct comparison. Indeed, these results are among the few available on benthic organisms (e.g., invertebrates such as molluscs, worms, crustaceans and echinoderms), which have been basically neglected so far [4]. Moreover, it is difficult to compare our results with other studies that were based on different experimental parameters (e.g., electrical field strength, frequency and exposure duration) and physiological alterations. Different studies have examined the effects of submarine power cable installation and operation on benthic communities. A slightly lower megafaunal density and a 100% glass sponge mortality were reported along cable transects [27]. Electric field exposures of 14 kV/m have been reported to negatively affect the viability of resting eggs and the juvenile survival of a freshwater ostracod *Heterocypris incongruens* [28]. On the other hand, no significant effects of electric field exposure have been found in zoobenthos species’ composition, abundance or biomass [10,29]. Similarly to our study, low current density values (0.4 and 0.8 μA/cm^2^) have been used to assess the response of crayfish (*Cherax destructor*) to an electric field. The results showed that crayfish alter their behavior in the presence of electrical fields in the surrounding water [23]. The effect of electrical fields on the viability of benthic fauna has also been evaluated in commercial electrofishing, again considering different experimental parameters (electric field strength, pulse current, frequency and exposure duration). Although electrical stimulation did not compromise the survival of the investigated species, several effects have been observed which are mainly associated with a change in locomotive behavior and development. Shelter behavior increased in flying crabs and hermit crabs after stimulation with a pulsed bipolar current of 200 V m^−1^ for 3 s [69]. An electric pulse led to a squirming reaction in ragworms and a tail flip response in shrimp, but also a virus infection when shrimp were stimulated at a higher current of 200 V m^−1^ [70]. A delayed hatching rate and decreased survival were observed for larvae of the Atlantic cod when exposed to a pulsed direct current of 150 V/m for 5 s [71]. Again, a negative effect on survival was found in ragworms, green crabs and common crabs [30]. The strongest negative behavioral reaction was observed in prawns and common crabs, and a weaker reaction in ragworms and razor clams [30].

## 5. Conclusions

The short-term exposure of the symbiont-bearing foraminiferal species *Amphistegina lessonii* to electrical current densities of both constant and pulsed current has deleterious effects on their pseudopodial activity. Pseudopodial activity was absent at high current densities (5.71 and 8.57 μA/cm^2^). The viability of A. lessonii exposed to a pulsed current was higher at low and middle electric current densities (from 0.29 to 5.71 μA/cm^2^) than at a high electric current density (from 11.43 to 20 μA/cm^2^). Our findings, therefore, suggest that *A. lessonii* specimens seem to better stand pulsed currents than constant ones. Indeed, it appears that a constant current has a more acute effect on viability than a pulsed current, even at low current densities and for shorter time exposures. These first experiments might provide useful information for the definition of an appropriate electrical density threshold to avoid side effects on a part of the benthic community and fill the knowledge gap of descriptor 11, “Introduction of energy, including underwater noise, is at levels that do not adversely affect the marine environment” of the Marine Strategy Framework Directive. Our results indicate the potential use of benthic foraminifera in environmental biomonitoring to evaluate the potential stress caused by artificial electric fields. Additional experiments, coupled with the detection of ultrastructural variations, enzymatic and protein pathway changes, are needed to better understand the physiological response of foraminiferal species to this poorly known form of anthropogenic impact.

## Data Availability

All data are fully available in Tables S1–S3.

## Supplementary Materials

The following supporting information can be downloaded at: https://www.mdpi.com/article/10.3390/life13040862/s1, Supplementary Material S1. Arduino Nano Code for pulsed stimulation. Supplementary Material S2. Arduino Nano Code for constant stimulation. Table S1: Summary of studies investigating the effects of artificial electric fields. Table S2: Raw data and percentages of individuals with pseudopodial activity in the symbiont-bearing foraminiferal species *Amphistegina lessonii* in samples treated with constant and pulsed current for 24 h, 48 h and 72 h and in control samples (no current). Table S3: Dunn’s test results showing the specific sample pair comparison.
