# Supplementary material for: Evaluation of the Effects of Electrical Stimulation: A Pilot Experiment on the Marine Benthic Foraminiferal Species Amphistegina lessonii
Source: Life (Basel). 2023 Mar 23;13(4):862. doi: 10.3390/life13040862 (PMC10142474; doi:10.3390/life13040862)
Supplement: Supplementary file 1 [file life-13-00862-s001.zip › life-2183618-supplementary.pdf]

Supplementary material S1. ArduinoNano Code for pulsed stimulation.

```
//www.diyusthad.com
#include <LiquidCrystal.h>

LiquidCrystal lcd(2, 3, 4, 5, 6, 7);

int analogPinV0 = A0;
int analogPinV1 = A1;
int analogPinV2 = A2;
int analogPinV3 = A3;
int analogPinV4 = A4;
int analogPinV5 = A5;
int analogPinV6 = A6;
int analogPinV7 = A7;

float val0 = 0;
float val1 = 0;
float val2 = 0;
float val3 = 0;
float val4 = 0;
float val5 = 0;
float val6 = 0;
float val7 = 0;

float current1 = 0;
float current2 = 0;
float current3 = 0;
float current4 = 0;

void setup() {

  Serial.begin(9600);
  pinMode (8, OUTPUT);
  pinMode (9, OUTPUT);
  pinMode (10, OUTPUT);
  pinMode (11, OUTPUT);
  pinMode (13, OUTPUT);

  lcd.begin(16, 2);
  lcd.print(current1);
  lcd.setCursor(3,0);
  lcd.print(current2);
  lcd.setCursor(0,1);
  lcd.print(current3);

  lcd.setCursor(8,0);
  lcd.print(current4);

}
```

```
void loop()
{
  while (1)
{
digitalWrite(8, HIGH);
digitalWrite(9, HIGH);
    digitalWrite(10, HIGH);
    digitalWrite(11, HIGH);
    digitalWrite(13, HIGH);

  val0 = analogRead(analogPinV0);
  val1 = analogRead(analogPinV1);
  val2 = analogRead(analogPinV2);
  val3 = analogRead(analogPinV3);
  val4 = analogRead(analogPinV4);
  val5 = analogRead(analogPinV5);
  val6 = analogRead(analogPinV6);
  val7 = analogRead(analogPinV7);
  //delay (1000);

  current1 = ((val0-val1)*0.0046)/0.047;
  current2 = ((val2-val3)*0.0046)/0.047;
  current3 = ((val4-val5)*0.0046)/0.047;
  current4 = ((val6-val7)*0.0046)/0.047;
  //10 kohm 0.01, 100 kohm 0.1

  //Serial.println(val5);

lcd.begin(16, 2);
lcd.setCursor(0,0);
lcd.print("1)");
lcd.setCursor(2,0);
lcd.print(current1);

lcd.setCursor(8,0);
lcd.print("2)");
lcd.setCursor(10,0);
lcd.print(current2);

lcd.setCursor(0,1);
lcd.print("3)");
lcd.setCursor(2,1);
lcd.print(current3);

lcd.setCursor(8,1);
lcd.print("4)");
lcd.setCursor(10,1);
lcd.print(current4);

Serial.println(current4);
```

```
 delay (1000);

  digitalWrite(8, LOW);
  digitalWrite(9, LOW);
    digitalWrite(10, LOW);
    digitalWrite(11, LOW);
     digitalWrite(13, LOW);

     delay (1000);

   //delay (500);
   }

 }
```

Supplementary material S2. Arduino Nano Code for constant stimulation.

```
//www.diyusthad.com
#include <LiquidCrystal.h>

LiquidCrystal lcd(2, 3, 4, 5, 6, 7);

int analogPinV0 = A0;
int analogPinV1 = A1;
int analogPinV2 = A2;
int analogPinV3 = A3;
int analogPinV4 = A4;
int analogPinV5 = A5;
int analogPinV6 = A6;
int analogPinV7 = A7;

float val0 = 0;
float val1 = 0;
float val2 = 0;
float val3 = 0;
float val4 = 0;
float val5 = 0;
float val6 = 0;
float val7 = 0;

float current1 = 0;
float current2 = 0;
float current3 = 0;
float current4 = 0;

void setup() {
  Serial.begin(9600);
  pinMode (8, OUTPUT);
   pinMode (9, OUTPUT);
    pinMode (10, OUTPUT);
```

```
    pinMode (11, OUTPUT);
     pinMode (13, OUTPUT);

  lcd.begin(16, 2);
 lcd.print(current1);
 lcd.setCursor(3,0);
 lcd.print(current2);
 lcd.setCursor(0,1);
  lcd.print(current3);

 lcd.setCursor(8,0);
 lcd.print(current4);

}

void loop() {
 while (1)
 {

 digitalWrite(8, HIGH);
  digitalWrite(9, HIGH);
   digitalWrite(10, HIGH);
    digitalWrite(11, HIGH);
     digitalWrite(13, HIGH);

  val0 = analogRead(analogPinV0);
 val1 = analogRead(analogPinV1);
 val2 = analogRead(analogPinV2);
 val3 = analogRead(analogPinV3);
 val4 = analogRead(analogPinV4);
 val5 = analogRead(analogPinV5);
 val6 = analogRead(analogPinV6);
 val7 = analogRead(analogPinV7);
 //delay (1000);

 current1 = ((val0-val1)*0.0046)/0.047;
 current2 = ((val2-val3)*0.0046)/0.047;
 current3 = ((val4-val5)*0.0046)/0.047;
 current4 = ((val6-val7)*0.0046)/0.047;
   Serial.println(val5);

  lcd.begin(16, 2);

  lcd.setCursor(0,0);
 lcd.print("1)");
 lcd.setCursor(2,0);
 lcd.print(current1);

  lcd.setCursor(8,0);
 lcd.print("2)");
 lcd.setCursor(10,0);
```

```
 lcd.print(current2);

 lcd.setCursor(0,1);
 lcd.print("3)");
 lcd.setCursor(2,1);
 lcd.print(current3);

 lcd.setCursor(8,1);
 lcd.print("4)");
 lcd.setCursor(10,1);
 lcd.print(current4);
 Serial.println(current4);

//delay (1000);
 //digitalWrite(8, LOW);
 //digitalWrite(9, LOW);
   //digitalWrite(10, LOW);
   //digitalWrite(11, LOW);
    //digitalWrite(13, LOW);
    //delay (1000);
   delay (500);
  }
}
```

Table S1. Summary of studies investigating the effects of artificial electric fields.

Authors	Organism	Electric induction	Observed effects
Hutchison et al 2018	American lobsters (H. americanus) and skat (L. erinacea)	AC cable (1-2.5 mV/m) DC cables (0.4-0.7 mV/m)	Slight change in behavioral activity in H. Americanus and strong behavioral response in L. erinacea
Love et al.2017	Crabs (M. magister, Cancer productus)	AC 35-69 kV	Change in the catchability
Love et al. 2015	Crabs (M. magister, Cancer productus)	46.2 mT to 80.0 mT	No effect
Patullo et al. 2007	Crayfish	0.4 and 0.8 μA/cm^2	Behavioral changes or reduction in body movement
Steullet et al. 2007	Crayfish	DC 20 mV/cm	Behavioral changes or reduction in body movement
Patullo Macmillian 2010	Crayfish	450 μV cm^{-1}, 300 μV cm^{-1}	Reduction in body motion
Kogan et al. 2006	Benthic communities	no mentioned	No effect
Bieszke et al 2020	Ostracod (Heterocypris incongruens)	14 kV/m	Negative effects on the viability of resting eggs and juvenile survival

Dhunam et al. 2015	Glass sponge reef and associeted megafaunal communities	230 kV HVAC cables	Glass sponge mortality and slight decrease in megafaunal abundance
Kuhnz et al. 2015	Macrofauna and Megafauna communities	no mentioned	Few potential changes in benthic communities
Van Maerlen et al 2009	Benthic invertebrates: (ragworm (Nereis virens L.), common prawn (Palaemon serratus L.), subtruncate surf clam (Spisula subtruncata L.), European green crab (Carcinus maenas L.), common starfish (Asterias rubens L.), and Atlantic razor clam (Ensis directus L.))	0.5 mV/A	Negative effect on survival in ragworm, green crab and common crab. Negative behavioural reaction in prawn and common crab, and a weaker reaction in ragworm and razor clam.
Tricas and New 1997	Elasmobranchs	5-20 nV m^{-1}	Acute sensitivity to electric fields
Kalmijn 1982	Elasmobranchs	0,005 µV cm^{-1}	Detection of weak stimulus
Kimber 2008	Elasmobranchs	400–600 µV m^{-1}	Avoidance behavior
Kimber et al. 2011	Elasmobranchs	~60 µV m^{-1}	No effect
Gill and Taylor 2011	Elasmobranchs	10 µV cm^{-1}	Avoidance behavior
Yano et al. 2000	Elasmobranchs	1,000 µV m^{-1}	Avoidance behavior
Walker 2001	Elasmobranchs	190 - 19000 µV cm^{-1}	Repulsion behavior
Giil et al. 2012	Teleosts fish	no mentioned	No effect on physiology or survival
Poddubny 1967	Sturgeon	110 kV	Avoidance behavior
Woodruff et al. 2013	Teleosts fish: Atlantic halibut (Hippoglossus hippoglossus), Crustacean species: Dungeness crab (Metacarcinus magister), American lobster (Homarus americanus)	1.0 - 1.2 mT (DC)	Few behavioral responsess
Berge 1979	European Eel	Weak AC and DC fields	Minimal and temporary effect
Westerberg and Lagenfelt 2008	European Eel	130 kV AC power cable	Lower swimming speed
Poleo et al 2001	Eel and Salmon	7 and 70 mV/m	Repulsion behavior

Table S2. Raw data and percentages of individuals with pseudopodial activity in the symbiont-bearing foraminiferal species *Amphistegina lessonii* in samples treated with constant and pulsed current for 24 h, 48 h and 72 h and in control samples (no current).

Constant current

Time	Current density µA/cm2	Number living specimens (pseudopodial activity)					Pseudopodial activity (%)
		1	2	3	4	5	
24h	Control	10	10	10	10	10	100%
	0,29	7	10	7	8	10	84%
	0,86	4	10	4	3	5	52%
	1,43	4	0	4	1	2	22%
	2,86	0	1	0	1	1	6%
	5,71	0	0	0	0	0	0%
	8,57	0	0	0	0	0	0%
48h	Control	10	10	10	10	10	100%
	0,29	5	9	5	4	5	56%
	0,86	0	3	5	2	3	26%
	1,43	0	0	0	0	0	0%
	2,86	0	0	0	0	0	0%
	5,71	0	0	0	0	0	0%
	8,57	0	0	0	0	0	0%
72h	Control	10	10	10	10	10	100%
	0,29	0	7	2	2	2	26%
	0,86	0	0	1	0	1	4%
	1,43	0	0	0	0	0	0%
	2,86	0	0	0	0	0	0%
	5,71	0	0	0	0	0	0%
	8,57	0	0	0	0	0	0%

Pulsed current

Time	Current density µA/cm2	Number living specimens (pseudopodial activity)					Pseudopodial activity (%)
		1	2	3	4	5	
24h	Control	10	10	10	10	10	100%
	0,29	10	10	10	10	10	100%
	0,86	10	10	10	10	10	100%
	1,43	10	10	8	10	10	96%
	2,86	10	10	8	10	10	96%
	5,71	9	10	8	9	9	90%
	8,57	8	10	4	7	8	74%
	11,43	0	0	0	0	0	0%
	14,29	0	0	0	0	0	0%
	17,14	0	0	0	0	0	0%
	20,00	0	0	0	0	0	0%

48h	Control	10	10	10	10	10	100%
	0,29	10	10	9	10	10	98%
	0,86	10	10	9	10	9	96%
	1,43	10	10	7	9	10	92%
	2,86	10	8	8	9	8	86%
	5,71	9	7	7	8	7	76%
	8,57	5	0	2	1	2	20%
	11,43	0	0	0	0	0	0%
	14,29	0	0	0	0	0	0%
	17,14	0	0	0	0	0	0%
	20,00	0	0	0	0	0	0%
72h	Control	10	10	10	10	10	100%
	0,29	9	9	9	10	9	92%
	0,86	9	9	9	9	8	88%
	1,43	5	9	5	6	7	64%
	2,86	5	6	4	6	5	52%
	5,71	2	4	7	5	4	44%
	8,57	0	0	1	1	0	4%
	11,43	0	0	0	0	0	0%
	14,29	0	0	0	0	0	0%
	17,14	0	0	0	0	0	0%
	20,00	0	0	0	0	0	0%

Table S3. Dunn's test results showing the specific sample pair comparison.

**Constant Current**	**24h**		**48h**		**72h**	
**Pair**	**Mean Rank difference**	**p-value**	**Mean Rank difference**	**p-value**	**Mean Rank difference**	**p-value**
0-1	3.3	0.5983	5.5	0.3366	7.7	0.1479
0-3	8	0.2016	11.5	**p<0.05**	15.3	**p<0.01**
0-5	14.8	**p<0.05**	22,00	**p<0.001**	20.5	**p<0.001**
0-10	19.4	**p<0.01**	22,00	**p<0.001**	20.5	**p<0.001**
0-20	24.5	**p<0.001**	22,00	**p<0.001**	20.5	**p<0.001**
0-30	24.5	**p<0.001**	22,00	**p<0.001**	20.5	**p<0.001**
1-3	4.7	0.4531	6,00	0.2945	7.6	0.1533
1-5	11.5	0.06639	16.5	**p<0.01**	12.8	**p<0.05**
1-10	16.1	**p<0.05**	16.5	**p<0.01**	12.8	**p<0.05**
1-20	21.2	**p<0.001**	16.5	**p<0.01**	12.8	**p<0.05**
1-30	21.2	**p<0.001**	16.5	**p<0.01**	12.8	**p<0.05**
3-5	6.8	0.2777	10.5	0.06658	5.2	0.3285
3-10	11.4	0.06878	10.5	0.06658	5.2	0.3285

3-20	16.5	**p<0.01**	10.5	0.06658	5.2	0.3285
3-30	16.5	**p<0.01**	10.5	0.06658	5.2	0.3285
5-10	4.6	0.4628	0	1,00	0	1,00
5-20	9.7	0.1215	0	1,00	0	1,00
5-30	9.7	0.1215	0	1,00	0	1,00
10-20	5.1	0.4156	0	1,00	0	1,00
10-30	5.1	0.4156	0	1,00	0	1,00
20-30	0	1	0	1,00	0	1,00

**Pulsed Current**	**24 h**		**48 h**		**72 h**	
**Pair**	**Mean Rank difference**	**p-value**	**Mean Rank difference**	**p-value**	**Mean Rank difference**	**p-value**
0-1	0	1	2.2	0.8207	6	0.5371
0-3	0	1	4.4	0.6504	8.5	0.3819
0-5	3.6	0.7012	6.2	0.523	15.8	0.1041
0-10	3.6	0.7012	11.8	0.2242	19.7	**p<0.05**
0-20	12	0.2009	17.4	0.07307	22	**p<0.05**
0-30	15.8	0.09217	26.5	**p<0.01**	35.5	**p<0.001**
0-40	32.5	**p<0.001**	36.5	**p<0.001**	40.5	**p<0.001**
0-50	32.5	**p<0.001**	36.5	**p<0.001**	40.5	**p<0.001**
0-60	32.5	**p<0.001**	36.5	**p<0.001**	40.5	**p<0.001**
0-70	32.5	**p<0.001**	36.5	**p<0.001**	40.5	**p<0.001**
1-3	0	1	2.2	0.8207	2.5	0.797
1-5	3.6	0.7012	4	0.6803	9.8	0.3134
1-10	3.6	0.7012	9.6	0.3227	13.7	0.1587
1-20	12	0.2009	15.2	0.1174	16	0.09976
1-30	15.8	0.09217	24.3	**p<0.05**	29.5	**p<0.01**
1-40	32.5	**p<0.001**	34.3	**p<0.001**	34.5	**p<0.001**
1-50	32.5	**p<0.001**	34.3	**p<0.001**	34.5	**p<0.001**
1-60	32.5	**p<0.001**	34.3	**p<0.001**	34.5	**p<0.001**
1-70	32.5	**p<0.001**	34.3	**p<0.001**	34.5	**p<0.001**
3-5	3.6	0.7012	1.8	0.8529	7.3	0.4527
3-10	3.6	0.7012	7.4	0.4459	11.2	0.2492
3-20	12	0.2009	13	0.1805	13.5	0.1649
3-30	15.8	0.09217	22.1	**p<0.05**	27	**p<0.01**
3-40	32.5	**p<0.001**	32.1	**p<0.001**	32	**p<0.001**
3-50	32.5	**p<0.001**	32.1	**p<0.001**	32	**p<0.001**
3-60	32.5	**p<0.001**	32.1	**p<0.001**	32	**p<0.001**
3-70	32.5	**p<0.001**	32.1	**p<0.001**	32	**p<0.001**
5-10	0	1	5.6	0.564	3.9	0.6883
5-20	8.4	0.3706	11.2	0.2486	6.2	0.5236
5-30	12.2	0.1935	20.3	**p<0.05**	19.7	**p<0.05**

5-40	28.9	**p<0.01**	30.3	**p<0.01**	24.7	**p<0.05**
5-50	28.9	**p<0.01**	30.3	**p<0.01**	24.7	**p<0.05**
5-60	28.9	**p<0.01**	30.3	**p<0.01**	24.7	**p<0.05**
5-70	28.9	**p<0.01**	30.3	**p<0.01**	24.7	**p<0.05**
10-20	8.4	0.3706	5.6	0.564	2.3	0.813
10-30	12.2	0.1935	14.7	0.13	15.8	0.1041
10-40	28.9	**p<0.01**	24.7	**p<0.05**	20.8	**p<0.05**
10-50	28.9	**p<0.01**	24.7	**p<0.05**	20.8	**p<0.05**
10-60	28.9	**p<0.01**	24.7	**p<0.05**	20.8	**p<0.05**
10-70	28.9	**p<0.01**	24.7	**p<0.05**	20.8	**p<0.05**
20-30	3.8	0.6855	9.1	0.3485	13.5	0.1649
20-40	20.5	**p<0.05**	19.1	**p<0.05**	18.5	0.05701
20-50	20.5	**p<0.05**	19.1	**p<0.05**	18.5	0.05701
20-60	20.5	**p<0.05**	19.1	**p<0.05**	18.5	0.05701
20-70	20.5	**p<0.05**	19.1	**p<0.05**	18.5	0.05701
30-40	16.7	0.07508	10	0.303	5	0.607
30-50	16.7	0.07508	10	0.303	5	0.607
30-60	16.7	0.07508	10	0.303	5	0.607
30-70	16.7	0.07508	10	0.303	5	0.607
40-50	0	1	0	1	0	1
40-60	0	1	0	1	0	1
40-70	0	1	0	1	0	1
50-60	0	1	0	1	0	1
50-70	0	1	0	1	0	1
60-70	0	1	0	1	0	1
